# Signaling Pathways and Potential Therapeutic Strategies in Cardiac Fibrosis

**DOI:** 10.3390/ijms24021756

**Published:** 2023-01-16

**Authors:** Alexandrine Bertaud, Ahmad Joshkon, Xavier Heim, Richard Bachelier, Nathalie Bardin, Aurélie S. Leroyer, Marcel Blot-Chabaud

**Affiliations:** Center for Cardiovascular and Nutrition Research, Aix-Marseille University, Inserm 1263, Inrae 1260, 27 Bd J. Moulin, 13005 Marseille, France

**Keywords:** cardiac fibrosis, heart failure, therapy, signaling pathways, biomarker, fibroblast

## Abstract

Cardiac fibrosis constitutes irreversible necrosis of the heart muscle as a consequence of different acute (myocardial infarction) or chronic (diabetes, hypertension, …) diseases but also due to genetic alterations or aging. Currently, there is no curative treatment that is able to prevent or attenuate this phenomenon that leads to progressive cardiac dysfunction and life-threatening outcomes. This review summarizes the different targets identified and the new strategies proposed to fight cardiac fibrosis. Future directions, including the use of exosomes or nanoparticles, will also be discussed.

## 1. Introduction

Cardiac fibrosis is a common feature of acute myocardial infarction (MI) and various other chronic diseases, such as hypertension, diabetes mellitus, and chronic kidney disease [1]. Heart failure (HF) is associated with a high mortality and poor quality of life and exerts a heavy burden on health systems. Epidemiological studies demonstrated that about six million American adults are suffering from HF, according to data from 2015 to 2018. HF incidence reaches 10 per 1000 in the population after 65 years. Numerous studies emphasized that the severity of cardiac fibrosis correlates with adverse cardiac events and mortality [2,3]. 

Cardiac fibrosis is defined as an increase in the myocardial extracellular matrix (ECM) protein deposition, mainly collagen I and III, that impairs cardiac function. Two types of cardiac fibrotic lesions have been defined depending on their localization and the feature of ECM protein deposition [4]. The first one is a reparative process, also named replacement fibrosis, that is observed as scar tissue. In this ischemic disease, oxygen deprivation of the heart muscle results in the necrosis and apoptosis of cardiomyocytes, leading to a loss of large amounts of cardiac cells that are essential for cardiac function. Cardiomyocyte death initiates a triphasic immune response that aims at clearing cell debris and promoting the replacement of the injured myocardium to maintain cardiac function [5]. The second type of fibrotic lesion is interstitial fibrosis, characterized by the diffuse deposition of collagen in the endomysium and perimysium. This interstitial fibrosis frequently comes with perivascular fibrosis and is specifically observed as secondary to chronic injuries, such as a pressure overload (aortic stenosis, hypertension), cardiac inflammation (myocarditis), and metabolic disorders (obesity, diabetes mellitus) as well as aging. Diffuse fibrosis is also frequently observed in the surviving infarcted heart, where it develops in remote areas. The myocardial interstitial fibrosis development alters myocardial architecture and physiology, modifying left ventricular compliance, diastolic function, and electrical connectivity, leading to arrythmia and adverse outcomes (hospitalization, mortality) [6,7,8]. Whatever the context, interstitial cardiac fibrosis is correlated with cardiac dysfunctions and is known to contribute to HF with or without preserved ejection fraction. Thus, understanding the molecular pathways involved in cardiac fibroblast activation will permit the development of new therapeutic strategies to fight cardiac fibrosis and reverse HF. 

This review summarizes the most relevant targets identified, their signaling pathways and the new strategies proposed to target them.

## 2. The Major Role Played by Cardiac Fibroblasts

Fibroblast activation and differentiation into myofibroblasts is a common process in all fibrosis-associated diseases. In response to cardiac injury, cardiac myofibroblasts are more proliferative and contractile and are stronger producers of extracellular matrix molecules and growth factors. Myofibroblasts are classically characterized by their increased proliferation rate and by the expression of multiple proteins such as α-smooth muscle actin (SMA), periostin, and collagen. Recent findings identified a subset of fibroblasts in the injured heart and demonstrated a differential pattern of expression, suggesting that several activated fibroblasts coexist and execute different roles [9]. Identifying the origin of the myofibroblasts responsible for the excessive ECM deposition takes a central place in the search for new tools to fight cardiac fibrosis. Multiple cell lineages have been proposed that contribute to activated cardiac fibroblasts. The activation of resident cardiac fibroblasts, as well as the differentiation of endothelial, epicardial, and perivascular cells (pericyte, vascular smooth muscle cells) or hematopoietic bone marrow-derived progenitor cells, have been explored in many models of heart injury [10,11]. For a long time, hematopoietic cells were also proposed to transdifferentiate into cardiac fibroblasts in response to ischemic injury. Different studies showed a rapid mobilization of fluorescent cells into the infarct area, reaching nearly 20% of the SMA-positive cells [12,13,14]. However, these results are controversial since the contribution of the bone marrow cells is not clear and will necessitate future investigations using well-characterized and purified cell populations in these transplantation models. The embryonic origin of cardiac cells has prompted researchers to investigate the contribution of epicardial cells in the recruitment of cardiac fibroblasts that are secondary to heart injury. Postnatally, cardiac remodeling is associated with a re-expression of fetal epicardial genes and expansion of the epicardial layer [15,16]. In contrast to prenatal development, lineage tracing approaches demonstrated that the Epithelial-Mesenchymal transition (EMT) reactivation of epicardial cells does not contribute to the myocardial pool of cells implicated in cardiac fibrosis. Nevertheless, epicardial activity takes an important part in the healing process. Indeed, after MI, epicardial cells secrete various growth factors (VEGFA, FGF2) that promote angiogenesis in the infarcted myocardium, which suggests a paracrine contribution of epicardial cells to cardiac fibrosis [17,18,19].

The trans-differentiation of endothelial cells into fibroblasts through Endothelial-Mesenchymal transition (EndoMT) has been involved in many fibrotic processes (e.g., sclerodermia, lung fibrosis, and kidney fibrosis). EndoMT is characterized by a loss of endothelial cell markers (PECAM-1, VE-Cadherin) while acquiring mesenchymal ones (αSMA, vimentin, collagen). The TGFβ pathways, including the canonical (smad-dependent) and non-canonical (smad-independent) signaling pathways, together with ET-1 and Wnt pathways, have been described as involved in EndoMT and have been observed in many fibrotic contexts. In the injured heart, many animal models, including the transverse aortic constriction model and the myocardial infarct or angiotensin-induced fibrosis models, have highlighted the implication of TGFβ-induced EndoMT in cardiac fibrosis development [9,20]. EndoMT contribution has been largely revisited by Moorre-Moris in a pressure overload model of cardiac fibrosis [15]. In this study, endothelial cells isolated from lesions induced by aortic constriction applied to VE-cadherin-*CreERT2*^+/−^ collagen1a1-*GFP*^+/−^ *Rosa-tdT*^+/−^ mice did not express collagen 1, αSMA or PDGFRα, and fibroblasts did not express VE-cadherin. A recent work based on an original approach has confirmed these results. This new approach, associating genetic fate tracing, confocal imaging, and single-cell RNA sequencing, has explored vascular cell (endothelial cells) and perivascular niche (VSCM and pericytes) response and contribution to cardiac fibrosis in a transverse aortic constriction (TAC) model. The authors of this study established the existence of several pools of fibroblasts in the heart but did not find any proof of endothelial cells contributing to the mesenchymal lineage after TAC. They concluded that resident fibroblasts appeared to be the major cell population implicated in the disease development [21].

## 3. Molecular Mechanisms Involved in the Development of Cardiac Fibrosis

- Growth Factors

Among the various growth factors, the transforming growth factor β (TGFβ), fibroblast growth factor (FGF), and platelet-derived growth factor (PDGF) are the best studied. Elucidating their contribution and the partners implicated in the signaling pathways is a major research aim to identify precise mechanisms that may permit to trigger specifically cardiac fibrosis.

✓ *TGFβ*

Oxidative stress, Toll-like receptor signaling, and pro-inflammatory cytokines have been demonstrated to induce TGFβ isoforms’ expression in fibrotic tissues. In the heart, the fibrotic effects triggered by members of the TGFβ family are predominantly attributed to TGFβ1, even if TGFβ2 and TGFβ3 are involved with differences in temporal expression and biological functions [22]. TGFβ1 is the most studied isoform and has been demonstrated to exert a central role in the fibroblasts’ conversion into myofibroblasts. TGFβ1 induces the expression of myofibroblast markers (periostin, SMA, proteoglycan, collagen) and pro-fibrotic growth factors (such as the connective tissue growth factor, CTGF), which, in turn, enhance the activation of fibroblasts into myofibroblasts. Moreover, TGFβ1 has also been suggested to regulate ECM remodeling by promoting MMP/TIMP imbalance [23]. TGF-β1 is secreted as a precursor (inactive form) in a complex composed of the precursor (inactive form) of the TGF-β1, latency-associated protein (LAP), and latent TGF-β1-binding proteins (LTBPs), mainly sequestrated by an extracellular matrix. The latent TGF-β1 complex, often associated with the ECM, is cleaved by a wide range of proteases, including matrix metalloproteinases (MMP 2, MT1-MMP, ADAMTS) and activated integrins, such as α_v_β_6_ and α_v_β_8_ [24,25,26]. In this process, a triad based on AngII/TGFβ/CTGF amplifies the cardiac fibroblast activation. AngII induces TGFβ1 and CTGF via p38MAPK, which activate and enhance the fibrotic response of fibroblasts [27].

TGFβ1 receptor and pathways mediating the activation of fibroblasts into myofibroblasts have been intensively investigated to develop TGFβ1-specific therapies. Once activated, TGFβ1 can induce canonical (smad-dependent) and non-canonical (smad-independent) pathways. In the canonical pathway, the binding of TGFβ1 to the type II receptor (TRII) promotes the phosphorylation of type I receptor (mainly ALK5) and the recruitment and activation of receptor-regulated Sma- and Mad-related proteins 2 and 3 (named smad2 and smad3, members of R-smads). Smad2 or smad3 are released from the receptor and bind to smad4 to form a heterotrimeric complex which translocates to the nucleus and activates downstream effectors (SMA, collagen I, fibronectin, TIMP) via the nuclear recruitment of the coactivators (CREB-binding protein or p300 and Fast-1). Two inhibitory smads (I-smad), named smad6 and smad7, prevent R-smad phosphorylation. In the non-canonical pathway, TGFβ1 induces the phosphorylation of TRI (ALK5) or/and TRII, which mediate the activation of PI3K/Akt, the RhoA-ROCK axis, and MAPK signaling cascades [28]. The smad2/smad3 signaling pathways play a central role in the regulation of the canonical effect mediated by TGFβ1. In adult mice, smad2 does not seem to play a critical role in cardiac repair, although it is required for embryonic development. Conversely, smad3 is essential to mediate the TGFβ1 effect during cardiac fibrosis. Indeed, smad3 deletion is associated with a lesser extent of cardiac lesions, and smad3-mediated activation by TGFβ1 has been shown to induce myofibroblasts’ proliferation, ECM deposition, as well as TGFβ1 expression [29,30,31]. A triad based on AngII/TGFβ/CTGF amplifies the cardiac fibroblast activation dependent on p38MAPK [27].

The cross-talk between TGFβ and Wnt/β-catenin pathways has been proposed to regulate cardiac fibrosis as Wnt inhibition prevents the TGFβ-induced transformation of cardiac fibroblasts into myofibroblasts, and the activation of the Wnt pathway in the absence of TGFβ fails to generate myofibroblasts [32]. The signaling network that associates the TGFβ and Wnt/β-catenin pathways are unclear. Glycogen Synthase Kinase-3β (GSK3) has been proposed to ensure the crosstalk between these two pathways. In the Wnt/β-catenin pathway, Wnt binding to Fz/LRP induces GSK3 phosphorylation and inactivation, thus preventing β-catenin degradation. Preserved β-catenin accumulates in the cytoplasm and translocates to the nucleus to activate the fibrotic response. GSK3β-specific deletion in cardiac fibroblasts results in excessive fibrosis and adverse ventricular remodeling post-MI [33,34]. In vivo, the specific deletion of cardiac fibroblast GSK3β induces increased cardiac fibrosis and myocardial dysfunction post-MI. In vitro, GSK3β was demonstrated to exert a direct regulation of the TGFβ1-smad3 signaling pathway through direct interaction with smad3. GSK3β deletion in cardiac fibroblasts results in smad3 hyper-activation, as evidenced by its increased phosphorylation [35,36]. 

Unlike TGFβ1, many BMPs have been shown to exert anti-fibrotic effects during cardiac injury, including BMP2, BMP7, and BMP9 [37,38,39,40,41,42]. In TAC-induced cardiac fibrosis, each recombinant BMP attenuates cardiac fibrosis. Surprisingly, rhBMP7 also facilitates reverse remodeling after pressure overload release, providing proof of a potential therapeutic benefit of BMP7-based treatment against cardiac remodeling [41]. The mechanisms involved in the anti-fibrotic activity of BMP are not completely characterized. BMP canonical signaling has been largely described. In this process, unlike TGFβ, BMP binding to TIR recruits TIIR and activates smad1/5/ (8)9 transcription factors. In vitro, BMP signaling attenuates a TGFβ-induced fibrotic response by decreasing smad3 activation and enhancing smad1/5 phosphorylation. The molecular actors that control the switch between BMP and TGFβ signaling are not well defined. Endoglin, a coreceptor of the TGFβ1 and BMP receptor, has been suggested as a novel target to induce the BMP anti-fibrotic effect, as endoglin neutralization limits cardiac fibrosis in the TAC model by increasing BMP9 cardiac levels and reducing smad3 activation [42,43].

Among regulators of the TGFβ pathway, ROS generation has been demonstrated to potentiate a TGFβ effect on cardiac fibrosis genes. NAD(P)H oxidase 4 (NOX 4), a subcellular hydrogen peroxide generating enzyme, is upregulated in response to TGFβ1, PDGF, and Angiotensin II. A positive feedback loop may exist between ROS and TGFβ1 where NOX4 induces TGFβ1 latent form conversion to its active form and TGFβ1 upregulates NOX4 activity. In addition to the increased bioavailability of active TGFβ1, NOX4 may also regulate smad3 activation and enhance JNK and p38 phosphorylation, promoting the activation of fibroblasts [44,45,46]. In non-cardiac fibrosis models, such as pulmonary and kidney fibrosis, it has been suggested that NOX4 and TGFβ have cooperative activities to enhance fibrotic genes. Among those mechanisms, NOX4-induced ROS may allow the stabilization of SMA fibers by deacetylase mobilization and promote ECM synthesis. The last mechanism may associate NOX4, mTOR1/2, the transcription factors yes-associated protein (Yap), and the transcriptional coactivator with a PDZ-binding motif (TAZ). Altogether, these partners, activated by TGFβ1, enable pro-fibrotic gene expression enhancement [47].

Noteworthy, more than 60 non-coding microRNA (miRNA) have been reported to be involved in cardiac fibrosis. Many of these miRNAs modulate the TGFβ1 signaling pathway either directly by inhibiting TGFβ1, TGF β1 receptors, or smad proteins or indirectly by enhancing the TGFβ1/smad3 signaling pathway, thus conferring a pro- or anti-fibrotic property [48]. The exosome-mediated transport of miRNA was suggested to induce remote fibrotic effects besides many other cytokines and Wnt molecules. A recent publication entitled “Role of exosomal miRNA in heart failure” addresses new findings on exosomes and miRNA [49].

✓ *PDGF*

The PDGF family is composed of cell division stimulators that bind as disulfide-bonded homodimers and heterodimers. Five different subunits (PDGF-AA, PDGF-AB, PDGF-AB, PDGF-C, and PDGF-D) exist, which interact with two receptors, PDGFRα and PDGFRβ. The binding of PDGF induces PDGFR phosphorylation and activates downstream pathways (JAK/STATs, PI3/AKT, RAS/MAPK). All PDGF members have been shown to be involved in the development of cardiac fibrosis. While the overexpression of PDGF-A and -C induces cardiac fibrosis, PDGF-A overexpression induces a severe fibrotic reaction with an increased infarct area, leading to lethal cardiac failure. PDGF-C overexpression induces focal fibrosis with moderate cardiac hypertrophy [50,51,52]. PDGF-D has also been demonstrated to be upregulated in the infarct area, and its expression in the non-infarct area of the injured myocardium indicates that PDGF-D may be involved in the progression of interstitial fibrosis in post-infarct myocardium. In vitro experiments showed that cardiac fibroblast treatment with PDGF-D upregulates TGFβ1 and collagen I expression. Positive feedback between PDGF-D and TGFβ may participate in the spreading effect of PDGF-D on cardiac fibrosis, suggesting that PDGF-D targeting may allow the limitation of cardiac fibrosis- extent to non-infarcted myocardium [53]. PDGF receptors’s blockade experiments demonstrated a specific involvement of PDGFRβ in the vascular maturation of healing infarcts to ensure scar maturation and the resolution of the inflammation [54]. Considering that PDGF-D is involved in the extent of cardiac fibrosis and amplifies TGFβ expression, the PDGF-D targeting therapy may appear as an attractive option to fight interstitial fibrosis. However, the effect of the PDGFRβ blockade on vessel maturation and scar remodeling suggests that all cardiac fibrosis etiology cannot be fought by this approach [55].

✓ *FGF*

The FGF family is composed of 22 members with pleiotropic effects ranging from physiological cardiac development to pathological disease [56]. The interaction between FGF, their cofactors (heparan sulfate, klothos), and FGF receptors (FGFR) induce the dimerization of FGFR and the initiation of downstream signaling pathways (MAPK, PKC, Src-associated, and PI3K/Akt pathways). During embryonic and postanal development, FGF appears to play a major role in cardiac morphogenesis and homeostasis by regulating cell commitment and cardiomyocyte proliferation and differentiation [57]. For example, FGF2 regulates cardiac angiogenesis as well as cardiomyocytes’ and fibroblasts’ proliferation. In vivo, the deletion or overexpression of FGF2 in transgenic animal models submitted to cardiac injury allowed the identification of various functions of FGF2 in cardiac fibrosis [58,59]. FGF2 modulates fibroblasts’ proliferation, collagen deposition, endothelial cells’ proliferation, and cardiomyocytes’ behavior. The exact mechanism through which FGF2 induces these effects is not yet known. However, the antagonistic effect of the two FGF2 isoforms and the relation between TGFβ and FGF have been characterized. In fact, two distinct isoforms of FGF2 with different molecular weights exist. In fibroblasts, a secreted low molecular weight FGF2 (Lo-FGF2, 18kDa) binds to FGFR in combination with the heparan sulfate and activates downstream signaling. It also inhibits the trans-differentiation of fibroblasts into myofibroblasts in response to TGFβ. On the contrary, the high molecular weight FGF2 (Hi-FGF2, 20kDa) is transported into the nucleus, which induces SMA accumulation, EDA-FN, and collagen expression. Thus, Hi- and Lo-FGF2 exert apparently opposite effects on the differentiation of fibroblasts into myofibroblasts. This suggests that Lo-FGF2 exerts beneficial effects after MI [57,58,59,60]. Additionally, studies using non-cardiac cells demonstrate that Erk1/2, as induced by FGF, is able to suppress TGFβ1-induced canonical and non-canonical (p38) pathways to regulate the fibroblasts’ activation [60].

✓ TNF

In experimental mice models of MI, the serum concentration of the soluble Tumor Necrosis Factor alpha (TNFα) was found to be significantly increased as compared to the control group. In addition, TNFα was potently upregulated in the infarct zone and adjacent borders. Numerous studies have highlighted the key role of TNFα in fibrogenesis using transgenic mice models with deleted or overexpressed TNFα/TNFR1 (TNFα receptor 1) or using specific TNFα stimulating antibodies [61]. Importantly, soluble and membranous TNFα levels are increased in the sera and heart of mice post-infarction, respectively. In the MI model, TNFα^−/−^ mice exhibited smaller fibrotic lesions in the heart, lowered inflammatory responses, reduced cardiac hypertrophy, and preserved cardiac functions. In fact, TNFR1 is the main receptor mediating TNFα effects [61,62,63]. First of all, TNFα acts on cardiac fibroblasts to induce the expression of pro-inflammatory cytokines such as MCP-1 and MCP-3, which facilitate the recruitment of inflammatory cells toward the cardiac tissue [64]. Secondly, TNFα exacerbates fibrotic responses by inducing MMPs and CTGF expression [65,66]. Thus, TNFα indirectly contributes to the accumulation of the extracellular matrix by inducing CTGF expression. In turn, CTGF exerts autocrine activity leading to collagen deposition by fibroblasts [34,35,61]. In addition, ROS, through the PI3Kγ and MAPK signaling pathways activation, increases MMP expression [67]. Interleukins (IL-1 family and IL-6).

Inflammation is initially a reparative process that acts to restore cardiac function in response to injury. In myocardial infarct, the acute inflammatory phase is induced by dying-resident cells and immune cell infiltration, which ensures cell debris clearance and the transition to a reparative phase where activated fibroblasts towards myofibroblasts secrete large amounts of ECM proteins that compensate for lost tissue and preserve cardiac function. The suppression and resolution of the inflammatory phase are essential to prevent the expansion of the reparative process to the non-infarct area of the myocardium. In heart failure patients, the chronic inflammation state highlighted by increased levels of inflammatory cytokines (TNFα, IL-1, IL-6) is correlated to the severity of the disease and is an independent predictor of mortality. In animal models, the contribution of inflammation to the cardiac fibrosis process has been demonstrated [4].

The interleukin-1 (IL-1) family includes pro-inflammatory and anti-inflammatory members. Among IL-1 family members, IL-1α and IL-1β have been demonstrated to be increased in the sera from MI patients [68]. In animal models of MI, mice deleted for IL-1, IL1 blocking strategies using an antibody, or the use of exogenous IL-1 receptor antagonist demonstrated reduced infarct areas with improved cardiac function [69,70]. IL-33, an interleukin-1 family member, mediates the shift in the inflammatory phase toward its resolution through IL-1R4 (ST2). The ST2/IL-33 system has been demonstrated to regulate IL-33 fibrotic effects. In this system, IL-33 is released from injured cardiac cells bound to the ST2L transmembrane receptor, preventing cardiomyocyte death. In response to cardiac injury, cardiac fibroblasts and cardiomyocytes are the main source of a soluble form of the IM-33 receptor named sST2. sST2 regulates ST2L/IL-33 signaling [71]. Increased levels of sST2 avoid IL-33/ST2L signaling and, as a result, hampers the cardioprotective effects of IL-33 and allows a pro-fibrotic response. On the basis of this regulatory mechanism, sST2 has been explored as a biomarker in decompensated HF [72]. 

Studies have also validated a direct correlation between cytokines of the IL-6 family and the size of the infarct zone, as well as prognosis in patients with MI [73]. The depletion of IL-6 in various animal models decreased cardiac fibrosis as induced by cardiac overload or experimental MI [74,75]. In vitro, the hypoxia-inducible cytokine IL-6 increases the fibroblasts’ proliferation and enhances both SMA and TGFβ expression, corresponding to the fibroblasts’ activation [76,77].

- Renin-Angiotensin-Aldosterone system (RAAS)

RAAS is best recognized for its role in the physiological regulation of blood pressure, volume, and sodium homeostasis. It is well established that the activation of RAAS plays an important role in the development and progression of cardiovascular diseases. In fact, the inhibition of Angiotensin II, a major actor in the RAAS system, significantly increases mice survival and regresses left ventricular remodeling in preclinical studies [78]. AngII is generated systemically but also locally in some tissues, including the heart, where it acts through classical and non-classical RAAS pathways. In the classical pathway, the conversion of angiotensinogen into Angiotensin I (AngI) by renin is followed by its conversion to angiotensin II by the angiotensin-converting enzyme (ACE) or chymase [79]. In cardiac fibroblasts, AngII binding to AT1 induces cardiac fibroblasts’ proliferation, oxidative stress, and elevated fibrotic potential. The pro-fibrotic effects of AngII are mainly attributed to the induced increase in TGFβ1 production. As mentioned earlier, TGFβ1 induces smad2/3 and Erk1/2 phosphorylation, leading to the increased fibroblast expression of pro-inflammatory cytokines, the deposition of ECM proteins, and proliferation. Tenascin-C (TNC), periostin, and osteopontin (OPN) are three downstream products of AngII-induced fibroblast activation [80]. In all animal models of cardiac fibrosis induced by AngII, the density of cardiac fibroblasts significantly increased while, in TNC-KO mice, the fibrotic lesions were diminished. One mechanism that may explain this result is that TNC-induced expression by AngII promotes TGFβ1 expression by cardiac fibroblasts [81,82]. Recent findings demonstrate an interaction between the Prrx1-Twist1-TNC loop and SMAD2/3 [83,84]. Likewise, TNC has also been suggested to mediate an AngII-induced pro-inflammatory phenotype. For instance, TNC expression induced by AngII enhances the secretion of pro-inflammatory cytokines (IL-6, TNFα) by macrophages in a mechanism involving integrin αvβ3 and FAK/NFκb [85]. In this axis, AngII/TNC/IL-6 promotes the development of pro-inflammatory macrophages and the expression of fibrosis-associated genes in the fibroblasts. In addition, OPN appears to directly affect fibrotic processes since OPN-deleted mice showed attenuated interstitial fibrosis in response to AngII, compared to wild-type mice [86]. Finally, some of the effects of AngII (e.g., IL-6 and collagen I production) were attributed to the generation of a reactive oxygen species (ROS) [87,88]. Indeed, fibroblast proliferation, collagen deposition, MMP, and OPN production in response to AngII stimulation were blocked using a NAD(P)H oxidase inhibitor [89]. 

Importantly, recent findings suggest that the mitogen-activated protein kinase (MAPK) pathway mediates AngII/AT1R effects. P38 MAPK, a member of MAPKs, has been shown to play a central role in promoting the expression of pro-fibrotic genes in fibroblasts [80]. It has also been found that the AngII/AT1R/MAPK pathway affects RAAS by modulating the ACE/ACE2 ratio with increased ACE expression and decreased ACE2. This dysregulated balance results in the upregulation of the pro-fibrotic AngII synthesis and a decrease in the synthesis of its antagonist Ang (1-7).

In the alternative RAAS pathway, small peptides (e.g., Ang (1-7), Ang (1-9)) are generated from Ang I and AngII by ACEII. Ang (1-7) binds to MasR and AT2 receptors and exerts anti-oxidant, anti-fibrotic, and anti-proliferative effects, suggesting Ang (1-7)-MasR as a protective pathway. ACEII also contributes to anti-fibrotic effects mediated by the proteolytic degradation of Ang I and Ang II. Unfortunately, the data concerning the modulation of the alternative RAAS pathway during cardiac fibrosis is limited. Indeed, the deletion of ACEII was associated with severe cardiac dysfunction, whereas the increased activation of the MASR-Ang (1-7)-ACE2 pathway decreases myocardial fibrosis induced by cardiac injury [90,91,92]. Moreover, in a mouse model of MI, the impaired ACEII function leads to increased infarct size. Finally, pharmacological studies demonstrated that MASR-ACEII-Ang (1-7) induction impedes myocardial fibrosis by inhibiting or alleviating oxidative stress and apoptosis [93,94]. Finally, some of the effects of AngII (e.g., IL-6 and collagen I production) were attributed to the generation of reactive oxygen species (ROS) [87,88]. Indeed, the fibroblasts’ proliferation, collagen deposition, MMP, and OPN production in response to AngII stimulation was blocked using NAD(P)H oxidase inhibitor [89].

- β-adrenergic system

The induced activation of the β-adrenergic system is a compensatory mechanism that develops in response to reduced cardiac function to increase cardiac output and maintain blood pressure. However, in the long term, the chronic β-adrenergic system activation becomes detrimental, inducing increased myocardial fibrosis. In cardiac tissue, β1, β2, and β3 adrenergic receptors were expressed. While the stimulation of β1 and β2 receptors induces chronotropic and ionotropic effects, β3-receptor stimulation exerts the opposed effects. The pro-fibrotic effects of β-adrenergic receptor activation have been demonstrated in vivo and in vitro. Adrenergic stimulation induces cardiac fibroblast proliferation, collagen expression, and the activation of fibroblasts into myofibroblasts mediated by p38MAPK and PI3K [4]. Under chronic stimulation, adrenergic receptors are desensitized by a mechanism that involves G-protein-coupled kinase (GRK) and β-arrestin. In HF patients, GRK2 is elevated and has been proposed to counteract cardiac fibrosis [95,96].

## 4. Emerging Techniques for the Detection of Cardiac Fibrosis

✓ Imaging techniques

Developing non-invasive and sensitive techniques that enable the determination of the precise repartition of fibrosis in the myocardium is a real challenge. The current gold standard techniques are based on invasive procedures where myocardial biopsies are analyzed for their collagen content to estimate cardiac fibrosis (picrosirius red coloration). Unfortunately, heart sampling is difficult, and repeated biopsies to evaluate patients’ prognosis or response to certain treatments is almost impossible. Noteworthy, myocardial biopsies do not represent the entire fibrotic lesion, leading to imprecise diagnosis. Therefore, improving imaging techniques is of particular interest for the early detection of fibrinogenesis or already established fibrotic lesions. Developing and advancing non-invasive methods will facilitate the assessment of treatment efficacy and patients’ follow-up. Indeed, many non-invasive imaging techniques such as ultrasound-based echocardiography (ECH), cardiovascular magnetic resonance (CMR) imaging, or positron emission tomography (PET) have been used to identify fibrosis [97] with undeniable limitations.

Echocardiography is commonly used as a first-line diagnostic procedure for cardiac fibrosis. It provides indirect information on the myocardial structure and function. In addition, this technique allows the identification of multiple organs’ dysfunction secondary to MI or myocardial remodeling [98]. Moreover, the use of two-dimensional and three-dimensional speckle tracking echocardiography (STE) to assess MI-induced fibrosis proved to have insufficient sensitivity, especially in patients presenting mild to moderate cardiac fibrosis [99]. These drawbacks illustrate the necessity to upgrade those approaches.

CMR imaging associated with a gadolinium-based contrast agent combined with T1 mapping has been demonstrated to be of large interest in the identification of cardiac fibrosis lesions and diffuse fibrosis. Late gadolinium enhancement (LGE) permits to qualitative detection of large scars but is unable to detect or quantify diffuse fibrosis. An indirect CMR technique, such as T1 mapping, allows a quantitative assessment of diffuse heart fibrosis by the determination of native T1, post-contrast T1, and extracellular cardiac volume (ECV). Measuring ECV has prognostic significance in non-ischemic myocardial fibrosis [98,99,100,101].

Positron-emission tomography (PET) coupled with CT has been extensively used to investigate non-specific parameters such as cardiac perfusion and viability. It also allows assessing the extent of cardiac damage and response to conventional therapies [102]. Various clinical trials demonstrated promising results of PET/CT in imaging fibrotic lesions [103,104]. Radio-theranostics, which combines PET imaging with targeted radionucleotide therapy, is a great example of how combinational techniques may enhance diagnostics. Of importance, identifying new molecular markers specific of cardiac fibrosis will enhance radio-labeled based imaging techniques as well as target the delivery of therapeutic agents toward fibrotic lesions. To date, several targets have been explored in animal models of pulmonary or myocardial fibrosis to detect selectively activated fibroblasts, MMPs, collagen, or LOX [103,105,106].

✓ Circulating biomarkers of cardiac fibrosis

The identification of circulating markers of cardiac fibrosis is of major interest in the management of the disease. Among the various advantages, circulating markers may offer a non-invasive alternative to myocardial biopsies that allow the stratification of patients in terms of the evolution of cardiac fibrosis, helping define therapeutic approaches and evaluating responses to treatments. To this end, it is necessary to identify non-invasive, easily available, and specific circulating cardiac fibrosis markers. As cardiac fibrosis is characterized by an imbalance in myocardial ECM proteins and manifested by an increase in collagen I and III deposition, collagen crosslinking, and the modulation of MMPs and TIMP profiles, extracellular matrix remodeling proteins and peptides have been largely explored for their potential role as biomarkers.

Collagen I and III are secreted as soluble triple helix procollagens and are submitted to the hydrolysis of N- and C-termini, releasing tropocollagen with PNP and PCP peptides. The tropocollagen molecules then associate together via covalent intermolecular interactions mediated by the lysyl oxidase enzyme (LOX). Increased collagen cross-linking provides a tensile strength which contributes to myocardial stiffness and impaired ventricular relaxation [6]. During the collagen degradation step, proteolytic enzymes break down the collagen and release carboxy-terminal telopeptides (C1TP). Indeed, MMP members, such as MMP1 collagenase, are secreted in their latent form and then become activated in extracellular spaces by proteases (such as cathepsin, MMPs), ROS, and other extracellular matrix components [104]. In tissues, MMP activity is modulated by their specific tissue inhibitors (TIMPs). Consequently, it can be difficult to interpret MMP/TIMP as an imbalance in terms of collagen degradation or synthesis activity. Indeed, elevated circulating levels of MMP1 and TIMP1 are associated with poor outcomes in HF, and the C1TP/MMP1 ratio is inversely associated with cardiac complications in HF patients [107].

The sensitivity and specificity of P1CP peptides and biological assays to evaluate cardiac collagen deposition are still unclear. The sera level of P1CP is increased in sera from patients suffering from hypertension and dilated cardiomyopathy. Moreover, circulating levels of P1CP have been demonstrated to predict adverse outcomes in patients with HF and preserved ejection fraction [108,109,110,111]. However, the correlation of P1CP with cardiac fibrosis extent has to be demonstrated, and it must be noted that P1CP circulating levels may be modulated by other non-fibrotic or non-cardiac processes. Assessing peptides associated with collagen synthesis alone does not seem to constitute a sufficient strategy to specifically detect cardiac fibrosis [112,113]. In a multiparametric approach combining PICP, PIIINP, cardiac fibrosis area determination, and imaging on dilated cardiomyopathy biopsies, a study reported that P1CP/LGE combination provided a sensitive tool to predict adverse cardiac outcomes and that patients positive for cardiac fibrosis presented higher levels of P1CP and LGE [112,113,114]. 

As LOX expression is increased during cardiac fibrosis, the relevance of assessing circulating LOX has been studied [115]. The myocardial upregulation of LOX is effectively observed in the sera from HF patients [116,117]. However, the lack of correlation between cardiac fibrosis imaging and LOX activity in patients’ sera failed to ensure the pertinence of this marker.

Galectin-3, a β-galactoside binding protein expressed by various cells (macrophages, neutrophils, vascular cells, adipocytes, renal cells) and known to be upregulated during cardiac fibrosis, has also been measured in blood samples from HF patients [118]. Indeed, an elevated concentration of Galectin-3 in the blood positively correlates with the HF level and appears to be predictive of cardiac complications, in particular mortality, in HF [119,120]. Unfortunately, few studies have performed an in-depth evaluation of the association between circulating Galectin-3 and ECM deposition in the myocardium. In addition, discrepancies in studies concerning the correlation between circulating galectin-3 and the extent of cardiac fibrosis were hindered, considering galectin-3 as a reliable biomarker of cardiac fibrosis [121].

Circulating ST2 has been mainly investigated in cardiac pathologies such as myocarditis, heart failure, hypertension, and myocardial infarct. Major results have been recently summarized by Dudek et al. [122]. In brief, sST2 biomarkers have been demonstrated as an independent predictive marker of adverse outcomes and mortality in heart failure and may help to stratify patients [123,124]. However, sST2 concentration does not allow the diagnosis of HF [125].

Finally, various miRNAs have been reported to be upregulated during cardiac fibrosis. These circulating miRNAs have been found to be stable and easily detectable in the patients’s blood [126]. Therefore, some miRNAs have been suggested to act as novel biomarkers of cardiac fibrosis but fail as useful biomarkers of left ventricular remodeling [127]. Among them, the upregulation of circulating miR-21 and miR-133a was associated with cardiac fibrosis but in a non-specific manner. In the Eperelone Post-Acute Myocardial Infarction HF Efficacy and Survival Study (EPHESUS), only miR-113a was reported as a potentially specific circulating miRNA [48,128]. Thus, in the next few years, the development of standardized protocols for miRNA isolation and the correlation of miRNA with myocardial collagen content is necessary to consider miRNA as a circulating biomarker of cardiac fibrosis.

## 5. Potential Therapeutic Strategies for the Treatment of Cardiac Fibrosis

✓ *TGFβ1 inhibitors*

Considering the central role of TGFβ1 in cardiac fibrosis, blocking the TGFβ1 signaling activity has been supposed to be a promising approach to fight fibrosis. Many strategies blocking TGFβ1 signaling pathways have been explored in animal models, including antibodies and chemical molecules. Anti-TGFβ1 strategies were non-conclusive. In fact, whereas anti-TGFβ1 antibodies, when injected in an animal model of cardiac fibrosis, were promising asthey decreased cardiac fibrosis and diastolic abnormalities, these results were not achievable in MI models of cardiac fibrosis due to an increase in animal mortality [129,130]. Molecular inhibition of the TGFβ1 receptor, ALK5, in pressure overload-induced fibrosis attenuated cardiac fibrosis development, collagen crosslinking, and improved diastolic function. However, these beneficial effects on cardiac function were counterbalanced by increased cardiac dilatation and mortality [131]. Conversely, the injection of a dominant-negative form of the soluble TGFβ type II receptor after MI significantly improved ventricular dilatation and cardiac function, attested by a decrease in the infarct size. This suggests that TGFβ-targeting therapy is a promising tool for treating HF post-MI [132]. Although therapies targeting TGFβ demonstrated anti-fibrotic potentials in preclinical models, hemorrhage and the infiltration of inflammatory immune cells into cardiac valves, aorta, and vessels were documented in response to pan-TGFβ neutralizing antibodies [133,134,135,136].

Pirfenidone, an oral anti-fibrotic drug approved for the treatment of idiopathic pulmonary fibrosis by decreasing TGFβ expression, has been suggested to display anti-fibrotic activity and cardioprotective effects [137,138,139,140,141,142]. It also decreases inflammatory processes by reducing the expression of cytokines and inhibiting the expression and formation of NLRP3 inflammasome. The efficacy and safety of Pirfenidone have been tested in a double-blind phase 2 clinical trial enrolling HF patients with preserved ejection fractions treated for one year (PIROUETTE trial). A significant reduction in cardiac fibrosis, determined by ECV measurement in CRM, was achieved, suggesting that pirfenidone may exert beneficial anti-fibrotic activity and that further trials are necessary to determine the clinical effectiveness and safety of pirfenidone in HF.

✓ *RAAS targeting*

RAAS system inhibitors are actually the main therapies used to control cardiac fibrosis. RAAS inhibitors include several classes of molecules as a function of their molecular target. Direct inhibition of angiotensin II production is obtained by renin inhibitors and angiotensin-converting enzyme inhibitors (ACE-I). Finally, ARB, also named Angiotensin II receptor blockers, are used to antagonize the AT1R receptor of angiotensin II. 

Although in vivo renin inhibitors attenuate pro-fibrotic responses observed during MI and pressure overload induced-cardiac fibrosis in mice models, these beneficial effects were not observed in human clinical studies [86,143,144]. Moreover, in the ASTRONAUT study, Aliskiren, an orally active renin inhibitor approved for the treatment of hypertension, did not reduce cardiovascular death or rehospitalization as compared to the placebo, demonstrating that renin inhibition does not attenuate HF [145]. ARB (Valsartan, telmisartan, losartan, Olmesartan) and ACE-I (Lisinorpil, Enalapril) also reduce fibrosis and cardiac hypertrophy in animal models of fibrosis. Nevertheless, only a few studies have investigated the protective effect of ARB or/and ACE-I against cardiac fibrosis and correlated cardiac function and outcomes to cardiac collagen content [135,146,147,148,149,150,151,152]. Generally, ARB, as well as ACE-I treatments, decrease cardiac fibrosis in hypertensive patients. However, in HFpEF patients, mortality and hospitalization were not reduced, suggesting that therapy targeting Angiotensin II is insufficient to reduce cardiac fibrosis [6,126,153,154].

In addition to RAAS, the natriuretic peptides system (NPs) has been shown to be activated in HF and to correlate with its severity [155,156]. The biological activity of NP inactivates natriuresis, diuresis, and vasodilation and inhibits RAAS. NPs are inactivated by excretion, internalization, and by neprilysin-mediated degradation. As NP inactivates RAAS, strategies to increase NPs levels have been developed. The first one was based on the intravenous injection of therapeutic natriuretic peptides (e.g., Carperitide, Nesiritide). However, the limitation of these drugs in long-term HF treatment relies on their intravenous mode of administration and on their short duration of action. Another approach to increasing the NP level emerged from neprilysin characterization. While neprilysin inhibitors demonstrated positive effects on the NP level, they failed to sustain blood pressure reduction. This disappointing observation was explained by the increased level of angiotensin induced by neprilysin inhibitors but led to the development of strategies associated with angiotensin II and neprilysin inhibition. The first one was based on ACE inhibitors such as Enapril associated with the neprilysin inhibitor (Omapatrilat). Unfortunately, Omapatrilat treatment of hypertensive patients was associated with an increased risk of angioedema as compared to ACE alone due to increased bradykinin levels and the additive inhibitory effect of neprilysin and ACE inhibitor on bradykinin degradation [157,158]. ARNI (angiotensin receptor/neprilysin inhibitor) constitutes a new class of pharmacological therapy that simultaneously increases NP circulating levels and blocks the angiotensin II type I receptor. Sacubitril/Valsartan (LCZ696) was approved by the European Medicine Agency and the United States Food Drug Administration with an indication for chronic HF patients. Numerous trials indicate that Sacubitril/Valsartan may be beneficial in patients with HF. The PARADIGM-HF (prospective comparison of ARNI with ACEI in heart failure patients) trials that enrolled 8399 patients with HF showed that Sacubitril/Valsartan reduces the risk of hospitalization by 20% in comparison to RAAS inhibitors. The extrapolation of survival data from this study estimates that the beneficial treatment would result in 1–2 years of increase in survival. Sacubitril/Valsartan treatment was well tolerated, and hypotension was the major observed adverse effect [159,160,161,162]. In heart failure patients, Sacubitril/Valsartan treatment was demonstrated to improve cardiac function and decrease myocardial hypertrophy, suggesting an effect on cardiac fibrosis [163,164,165]. There is a lack of proof concerning the effect of RNA interference (RNAi) treatment on cardiac fibrosis in the myocardium of patients. The measurement of a panel of fibrosis-associated biomarkers demonstrated that RNAi treatment reduced significantly sST2, MMP-9, TIMP-1, and PINP in comparison to Enapril treatment, suggesting a beneficial effect of Sacubitril/valsartan on cardiac fibrosis in HF patients [166]. The demonstration of Sacubitril/Valsartan’s effectiveness on the cardiac fibrosis process in animal studies allowed the involved molecular pathways to be unraveled [167]. In the myocardial infarction model, Sacubitril/Valsartan treatment reversed the cardiac dysfunction induced by MI in comparison to non-treated animals and exerted a superior beneficial effect compared to Valsartan alone. The improved cardiac function induced by Sacubitril/Valsartan was associated with a decreased collagen volume fraction and collagen I and III contents in the myocardium [167,168,169]. In animal models of pressure overload-induced cardiac fibrosis, LCZ696 prevented cardiac hypertrophy and improved cardiac function [170,171]. The signaling pathways involved in the anti-fibrotic effect of LCZ696 have been partially elucidated and reviewed by Mustafa et al. in 2022. LCZ696 has been demonstrated to modulate fibroblast proliferation, SMA, collagen, CTGF, TGF, and MMP expression. This effect may be achieved through TGFβ pathway inactivation by decreasing smad3 activation and increasing inhibitory-smad 7 expressions [172,173,174,175]. The LCZ696 effect on cardiac fibrosis may also be mediated by its repressive activity on the wnt/βcatenin pathway, as observed in animal models of cardiac infarction and in cultured cardiac fibroblasts. The reduced collagen expression induced by the drug treatment is associated with a decreased β-catenin expression [176]. 

Aldosterone inhibition by the mineralocorticoid receptor antagonist proved its efficacy to decrease fibrosis and improve cardiac function in animal models without affecting blood pressure [177,178]. The administration of spironolactone to HF patients improved diastolic functions but failed to significantly reduce the incidence of hospitalization and death in HFpEF patients [137]. A study devoted to HFpEF suggested that spironolactone decreases diffuse myocardial fibrosis and indicated that MR therapy might have interest only in subgroups of patients [179,180].

## 6. Conclusions and Perspectives

**Figure 1:**
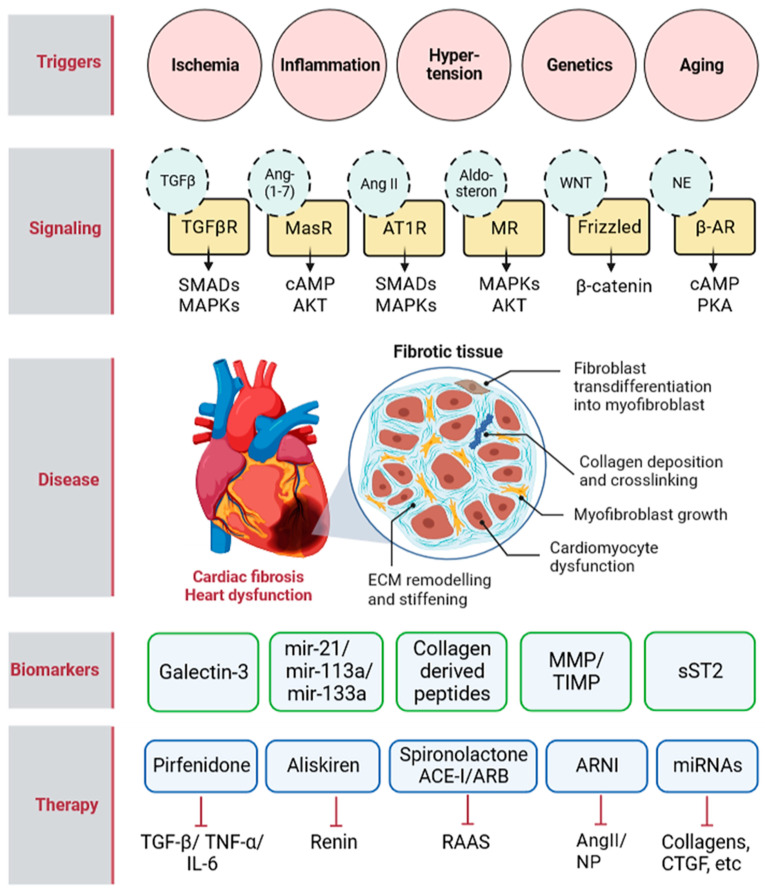
**Illustrative summary of cardiac fibrosis triggers, signaling pathways, biomarkers, and therapies.** Triggers of various origins alter the expression of multiple cytokines, hormones, and signaling peptides. This subsequently activates different signaling pathways which ultimately increase collagen deposition and crosslinking in the ECM, promotes the trans-differentiation of fibroblasts into myofibroblasts, and the proliferation and growth of myofibroblasts. The increase in ECM stiffness adversely affects the physiological function of cardiomyocytes which may lead to heart failure. Several biomarkers have been suggested to predict cardiac fibrosis and/or treatment efficiency. These include galectin-3, mir21, mir133a, mir113a, collagen derived peptides, MMP, TIMP, and soluble ST2. The currently available treatments include Pirfenidone which downregulates the expression of fibrogenic mediators such as TGF-β, TNF-α, and IL-6; Aliskiren inhibits renin; Spironolactone and ACE-I/ARB block RAAS; and miRNA are delivered using nanoparticles in order to target and destabilize mRNA of many fibrotic mediators. AT1R—angiotensin type 1 receptor; MR—mineralocorticoid receptor; NE—norepinephrine; β-AR—beta adrenergic receptor; MMP—matrix metalloproteinase, TIMP—tissue inhibitor of matrix metalloproteinase; sST2—soluble ST2; ARNI—angiotensin receptor-neprilysin inhibitor.

Cardiac fibrosis has become a major public health issue that necessitates improvements in therapy development. Research on cardiac fibrosis has evolved during the last two decades, and many new molecular actors have been identified thanks to the progress in genetics and proteomics approaches. In addition, many of these new targets have been demonstrated to be of therapeutic interest in animal models of cardiac fibrosis (Figure 1). However, molecular targets that have been identified are expressed in many non-cardiac tissues and may exert numerous adverse effects. Exosomal delivery of non-coding RNAs and proteins is a promising strategy to target cardiac tissue. Exosomes have demonstrated advantages, including small size, deformable structure, and capacity to escape from immunogen clearance. Moreover, their potential for delivering drugs or therapeutic molecules has been successfully tested in cancer [181]. Along this line, future research is needed to study their biodistribution and to ensure their selective delivery to cardiac cells in order to treat cardiac fibrosis. Another limitation is that biomarkers and imaging methods to detect cardiac fibrosis are insufficiently specific and sensitive. Therefore, developing new tools that allow both an early detection of cardiac fibrosis and the determination of its origin and characteristics will potentially lead to the rapid and efficient treatment of patients.

## Abbreviations

ACE	Angiotensin Converting Enzyme
Ang	Angiotensin
β-AR	Beta Adrenergic Receptor
ARB	Angiotensin-Renin Inhibitors
ARNI	Angiotensin Receptor-Neprilysin Inhibitor
AT1R	Angiotensin Type 1 Receptor
BMP	Bone Morphogenetic Protein
C1TP	Collagen 1 carboxy-Terminal Telopeptides
CMR	Cardiovascular Magnetic Resonance
CTGF	Connective Tissue Growth Factor
ECM	Extracellular Matrix
ECV	Extracellular Cardiac Volume
EndoMT	Endothelial to Mesenchymal Transition
ET-1	Endothelin-1
FGF	Fibroblast Growth Factor
GSK3	Glycogen Synthase Kinase-3β
HF	Heart Failure
HFpEF	Heart Failure with preserved Ejection Fraction
LAP	Latency-Associated Protein
LGE	Late Gadolinium Enhancement
LOX	Lysyl Oxidase enzyme
LTBP	Latent TGF-β1-Binding Proteins
MI	Mycocardial Infarction
MMP	Matrix Metalloproteinase
MR	Mineralocorticoid Receptor
NE	Norepinephrine
NOX	NADPH Oxidase
OPN	Osteopontin
PDGF	Platelet-Derived Growth Factor
PET	Positron Emission Tomography
RAAS	Renin Angiotensin Aldosterone System
ROS	Reactive Oxygen Species
SMA	α Smooth Muscle Actin
sST2	soluble ST2
STE	Speckle Tracking Echocardiography
TAC	Transverse Aortic Constriction
TGF	Transforming Growth Factor
TIMP	Tissue Inhibitor of Matrix Metalloproteinase
TNC	Tenascin-C
TRI	TGFβ1 Receptor Type I
TRII	TGFβ1 Receptor type II
